# ADVANCING TECHNOLOGY OF A GROUP MUSIC INTERVENTION FOR PERSONS WITH DEMENTIA: FINDINGS FORM A FOCUS GROUP STUDY

**DOI:** 10.1093/geroni/igad104.3788

**Published:** 2023-12-21

**Authors:** Silvia Orsulic-Jeras, Gregg Gorzelle, Michael Skrajner, Sara Powers, Sarah Nicolay

**Affiliations:** Benjamin Rose Institute on Aging, Cleveland, Ohio, United States; Hopeful Aging, Hiram, Ohio, United States; Hopeful Aging, Bolivar, Ohio, United States; Benjamin Rose Institute on Aging, Cleveland, Ohio, United States; Benjamin Rose Institute on Aging, Cleveland, Ohio, United States

## Abstract

The benefits of using music to engage persons living with dementia (PLWD) are widely reported in the literature Music can elicit memories and positive feelings as well as decrease anxiety and agitation in PLWD. Moreover, conducting music interventions in a group setting has been shown to increase social interaction, produce positive mood, and promote feelings of belonging. This paper discusses the adaptation and technological development of a group music intervention for persons living in the mild to moderate stages of dementia, Making Connections through Music (MCTM). Originally, MCTM was comprised of 6 individually themed sessions containing songs and corresponding discussion prompts; materials such as handheld instruments, photos, and props were used to elicit engagement and increase socialization among group members. Session leaders, older adult volunteers, were trained to provide the experience by offering the necessary structure and support PLWD need for successful participation. This paper will focus on the technological adaptation of the original MCTM intervention. Prototypes of a tablet application and online training course for volunteers were evaluated across seven focus groups with LTC staff (n=25) and older adult volunteers (n=25). Results from focus groups demonstrated 95% of volunteers and 100% of staff were satisfied with both the tablet app and training course. Acceptability and feasibility findings will be presented. Discussion also will highlight the implementation of this program within senior centers, adult day, and residential care as well as next steps in technological advancement of this intervention and associated training.

